# How epigallocatechin gallate binds and assembles oligomeric forms of human alpha-synuclein

**DOI:** 10.1016/j.jbc.2021.100788

**Published:** 2021-05-18

**Authors:** Camilla B. Andersen, Yuichi Yoshimura, Janni Nielsen, Daniel E. Otzen, Frans A.A. Mulder

**Affiliations:** 1 Interdisciplinary Nanoscience Center (iNANO), Aarhus University, Aarhus C, Denmark; 2 Department of Chemistry, Aarhus University, Aarhus C, Denmark; 3 Department of Molecular Biology and Genetics, Aarhus University, Aarhus C, Denmark

**Keywords:** alpha-synuclein (α-synuclein), NMR, protein aggregation, oligomer, oligomerization, stoichiometry, kinetics, αSN, α-synuclein, DSS, 2,2-dimethyl-2-silapentane-5-sulphonic acid, EGCG, (−)-epigallocatechin gallate, HSQC, heteronuclear single quantum coherence, NAC, non-amyloid β, STD, saturation transfer difference, TEM, transmission electron microscopy

## Abstract

The intrinsically disordered human protein α-synuclein (αSN) can self-associate into oligomers and amyloid fibrils. Several lines of evidence suggest that oligomeric αSN is cytotoxic, making it important to devise strategies to either prevent oligomer formation and/or inhibit the ensuing toxicity. (−)-epigallocatechin gallate (EGCG) has emerged as a molecular modulator of αSN self-assembly, as it reduces the flexibility of the C-terminal region of αSN in the oligomer and inhibits the oligomer's ability to perturb phospholipid membranes and induce cell death. However, a detailed structural and kinetic characterization of this interaction is still lacking. Here, we use liquid-state NMR spectroscopy to investigate how EGCG interacts with monomeric and oligomeric forms of αSN. We find that EGCG can bind to all parts of monomeric αSN but exhibits highest affinity for the N-terminal region. Monomeric αSN binds ∼54 molecules of EGCG in total during oligomerization. Furthermore, kinetic data suggest that EGCG dimerization is coupled with the αSN association reaction. In contrast, preformed oligomers only bind ∼7 EGCG molecules per protomer, in agreement with the more compact nature of the oligomer compared with the natively unfolded monomer. In previously conducted cell assays, as little as 0.36 EGCG per αSN reduce oligomer toxicity by 50%. Our study thus demonstrates that αSN cytotoxicity can be inhibited by small molecules at concentrations at least an order of magnitude below full binding capacity. We speculate this is due to cooperative binding of protein-stabilized EGCG dimers, which in turn implies synergy between protein association and EGCG dimerization.

The intrinsically disordered human protein α-synuclein (αSN) accumulates in the brains of patients with Parkinson's disease as intracellular deposits called Lewy bodies (1, 2). Self-assembly of αSN also occurs *in vitro*, where the protein is found to form oligomeric species and amyloid fibrils (3). The soluble oligomeric form that is formed by self-assembly is cytotoxic, in the sense that it binds and permeates cell membranes (4, 5, 6, 7, 8, 9). Recently, it was shown that monomeric αSN and iron-induced oligomeric forms of αSN interact in different ways with lipid membranes: whereas monomers cause membrane thinning and oligomers interact with and lead to changes within lipid raft–like domains (10). Different types of oligomers have been identified; some are thought to be obligate intermediates in fibril formation, whereas others are off pathway, forming independently and even inhibiting fibrillation (11, 12, 13). For example, an off-pathway oligomer can readily be formed by shaking αSN solutions at 37 °C. This oligomer consists of 30 protomers that form a compact β-sheet core surrounded by a diffuse corona (14, 15).

Given the demonstrated toxicity of oligomeric αSN, there is great interest in compounds that prevent their formation, or, once formed, reduce their toxicity. One such compound is (−)-epigallocatechin gallate (EGCG). Although EGCG typically shows low affinity for folded proteins (16), it binds numerous flexible proteins with no obvious sequence discrimination (17). Thus, EGCG binds monomeric αSN, affecting the entire amino acid sequence (16, 18), and also inhibits the fibrillation of several other proteins (19, 20, 21, 22, 23, 24). EGCG reduces the toxicity of αSN aggregates by remodeling amyloids into smaller nontoxic oligomers (22), redirecting the aggregation of monomeric αSN into nontoxic oligomers (8, 16, 18) and inhibiting the toxicity of preformed toxic αSN and amyloid-β oligomers *in vitro* (25, 26). Both solid-state (8) and liquid-state (25) NMR spectroscopy have demonstrated that EGCG-induced oligomers have altered structures compared with “naked” oligomers. Whereas naked oligomers insert into and disrupt the membrane (possibly in association with interaction between protein and lipids and conformational changes to the oligomer (27, 28)), EGCG-induced structural changes lead to reduced affinity for and an inability to perturb membranes (8). To complicate matters, αSN can form multiple different species of coexisting oligomers (13, 29). This makes it difficult to study the effect of EGCG on the interaction between oligomeric and monomeric αSN. As a result, we still lack a detailed structural and kinetic characterization of the interaction between EGCG and αSN in the process of oligomer formation. Obtained under *in vitro* conditions to obtain maximal structural insight, such advances provide a foundation to better understand how these oligomers subsequently may interact with membranes and other components in the cell.

Here, we use liquid-state NMR spectroscopy (30, 31) to show that EGCG binds to monomeric and oligomeric αSN. We find that monomeric and oligomeric αSN bind 54 and 7 EGCG molecules, respectively, and that EGCG slowly (over a 4-day period) binds to and immobilizes all protein residues. We suggest that these binding properties may explain the broad spectrum of activity by EGCG toward cytotoxic protein oligomers, and that related polyphenols act to bind together disordered proteins in a comparable way.

## Results

### EGCG redirects αSN monomer into oligomers in a dose-dependent manner

Determination of the stoichiometry of EGCG binding to αSN monomer by conventional titration is complicated by the fact that αSN oligomerizes in a time-dependent manner when EGCG is present (8, 16). Therefore, separate samples of αSN monomer were incubated with different amounts of EGCG, making sure to have identical incubation time (10 min) before recording 1D ^1^H-NMR spectra. Changes in the NMR spectra of both EGCG and αSN are expected upon interaction (for EGCG NMR assignments, see Fig. S1). These changes should manifest themselves as line broadening or chemical shift perturbation, with the exact outcome depending on binding kinetics (30). We hypothesized that our observations could be captured by a minimal model that considers (1) free and bound states for the protein as well as for EGCG and (2) that broadening of the NMR signals is caused by the modulation of the chemical (and magnetic) environment of the nuclei in the limiting states (free and bound), although more complex models can be envisaged (*e.g.*, intermediate exchange line broadening intrinsic to the bound form of EGCG and influence of EGCG on the self-association state of αSN). In addition, once oligomers of high molecular weight have formed, even a small population of the bound form in a weak-affinity interaction can bring about severe line broadening (32).

**Figure 1:**
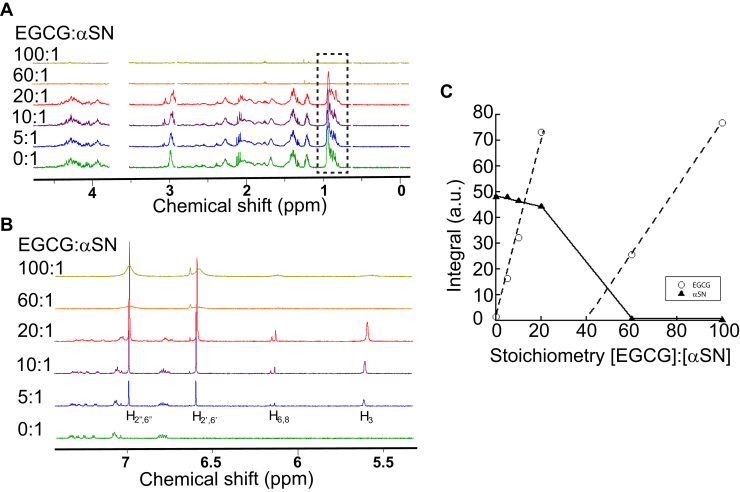
**EGCG induces αSN oligomerization.***A* and *B*, 1D ^1^H-NMR of αSN and EGCG at different stoichiometries. *A*, the methyl region from αSN (*dotted box*). Signals because of a glycerol impurity from protein concentration filter (3.6 ppm) and DSS (0.0, 0.625, and 2.9 ppm—but not the one at 1.75 ppm) have been removed to simplify the spectra (the unedited spectra can be seen in Fig. S2). *B*, the aromatic region (dominated by EGCG—H_2”,6”_, H_2’,6’_, H_6,8_, and H_3_ are assigned). *C*, integration of peak from EGCG H_2’,6’_ (at 6.6 ppm, see *B*) and integration of αSN methyl peaks (1.05–0.75 ppm). The *dashed line* and *solid line* in the figure are intended to guide the eye. αSN, α-synuclein; DSS, 2,2-dimethyl-2-silapentane-5-sulphonic acid; EGCG, (−)-epigallocatechin gallate.

Figure 1*A* displays the aliphatic region of 1D ^1^H-NMR spectra at different EGCG:αSN ratios. The region (2.5–0.75 ppm) shows signals from aliphatic side chains (mostly methyl groups) of αSN (EGCG does not show any resonances here, see Fig. S1) and is used to gauge the fate of the protein in solution. In contrast, the aromatic region (7.5–5.5 ppm) is dominated by EGCG (in *red boxes* in Fig. 1*B*), although there are weak signals from αSN's four Tyr and two Phe residues around 7.4 to 6.8 ppm (see lowest trace in Fig. 1*B*). Strikingly, using 10-min incubation, all protein signals fully disappeared when the [EGCG]:[αSN] ratio was 60:1 (Fig. 1, *A*–*C*), whereas only a very modest decrease was observed for αSN signals when titrating in the first 20 equivalents of EGCG. At the same time, EGCG NMR signals initially increased linearly with concentration up to 20 EGCG per αSN, in proportion to the amount of EGCG added, but upon an increase from 20:1 to 60:1, also all the EGCG signals vanished (Fig. 1, *A*–*C*). Since we did not see any precipitation, this implies that at ligand-to-protein ratios in the range 20 to 60, all EGCGs become sequestered in large, soluble, or dispersed assemblies that simultaneously cause both αSN and EGCG signals to become NMR invisible. This is the kind of behavior expected for molecules in a slow tumbling complex. Negative-stain EM demonstrated that loss of the NMR spectrum coincided with the formation of oligomeric species of ∼20 nm diameter (16).

Upon further addition of EGCG, αSN continues to remain NMR invisible, whereas a re-emergence of visible EGCG NMR signals is seen (Fig. 1*C*). The EGCG signals scale with the amount added, indicating accumulation of unbound EGCG. Consistent with this explanation, the line width of the EGCG peak for H_2’_,_6’_ is very little affected up to [EGCG]:[αSN] = 20:1. This indicates that, over the course of a 10-min incubation, most EGCG molecules exist in solution, and are only little affected by the presence of αSN, until a critical ratio is reached beyond 20 EGCGs per αSN. Once the NMR-invisible species have been fully formed, the signals of free ligand once more increase in intensity with further EGCG addition, but the peaks are very wide (Fig. 1, *B* and *D*). Broad peaks observed for small molecules typically point to the existence of a dynamic equilibrium between a rapidly tumbling free state and a slowly tumbling bound state (30). As ^1^H-NMR spectra of free EGCG consistently show sharp signals over the concentration range (0.15–3 mM) used in the αSN titration study (Fig. S3), we can rule out that broad signals result from self-association of EGCG (33, 34). This is in keeping with a reported EGCG self-association constant of 0.014 mM^−1^ (*i.e.*, 50% associated at 7 mM) at pH 6.0 (35).We conclude that a stoichiometrically well-defined EGCG:αSN complex is formed in the presence of a critical number of EGCG molecules.

### Time-dependent oligomerization of αSN in the presence of EGCG

**Figure 2:**
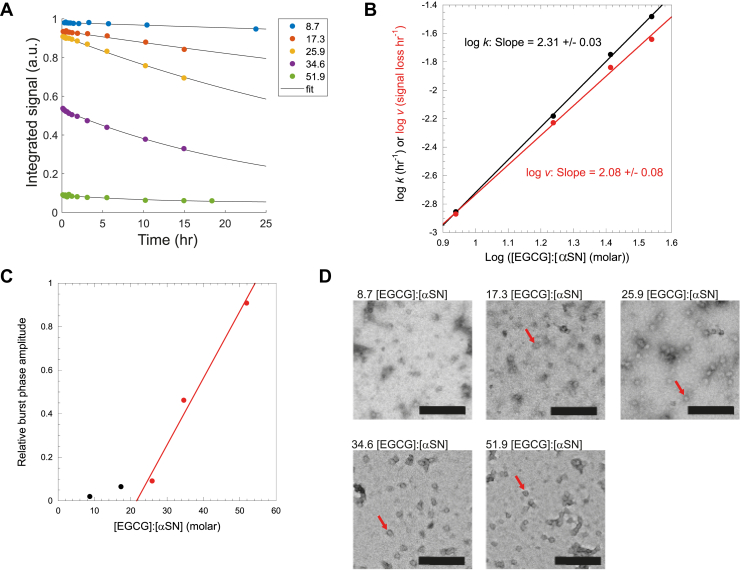
**Oligomerization of αSN in the presence of EGCG over time.***A*, EGCG-induced oligomerization of αSN, followed by integration of αSN peaks (2.5–0.6 ppm) in 1D ^1^H-NMR spectroscopy. Molar ratio [EGCG]:[αSN] and color code are provided alongside. *B*, growth rate from fit seen in *A* as a function of [EGCG]:[αSN] in a log–log plot. *C*, burst phase amplitude as a function of [EGCG]:[αSN] stoichiometry. *Red line* is a linear fit to the three data points at highest stoichiometry. The intercept at *y* = 1.0 occurs at a stoichiometry of ∼54. *D*, TEM images of αSN with EGCG taken after the NMR experiments. *Red arrows* are pointing at oligomers, and the *black* scale bar indicates 200 nm. αSN, α-synuclein; EGCG, (−)-epigallocatechin gallate.

To obtain mechanistic insight into the self-assembly process, we next investigated the time-dependent structural changes that take place during coincubation. To address this, EGCG and αSN were mixed at EGCG:αSN molar ratios ranging from 8.7 to 51.9, and a series of NMR spectra were recorded over time. A marked decline was observed for all 1D ^1^H-NMR signals (Fig. 2*A*), whereas no such decline in signals was seen in a control experiment (*i.e.*, in the absence of EGCG; data not shown). Using the integrated intensity of αSN protein NMR signals (2.5–0.6 ppm), the signal loss could be fitted using an exponential decay function (assuming that all signal is eventually lost), giving a rate constant *k*, which increased in a power-law fashion with EGCG:αSN stoichiometry up to 34.6 (Fig. 2*B*) (at higher stoichiometries, the signal change was too small to provide a reliable estimate of the rate constant). The order of a chemical reaction with regard to a given reactant can formally be determined by plotting the logarithm of the rate *v* (estimated from the initial slope in Fig. 2*A*) *versus* the logarithm of the concentration of that reactant. Whether using *k* or *v*, we obtain a slope of ∼2, which indicates that two EGCG molecules associate per reaction step, suggesting an EGCG dimerization associated with the mechanism of interaction with αSN. We note that at neutral pH, EGCG dimerizes (on the minute–hour scale) because of oxidation (36, 37), and dimeric EGCG has been shown to possess a more potent disaggregating effect than monomeric EGCG (38). Dimerization is possible, but not conclusively demonstrated by these data, which provide a lower limit for cooperativity of the EGCG–αSN interaction.

Interestingly, this slow signal decay was preceded by a very rapid loss of signal within the dead time of measurement (6–18 min) corresponding to a burst phase. The magnitude of this burst phase increased linearly with EGCG stoichiometry from 25.9 to 51.9 EGCG, and this linear phase extrapolated to the upper limit of 1.0 (*i.e.*, complete disappearance of the αSN methyl signal) around 54 [EGCG]:[αSN] (Fig. 2*C*). Assuming that rapid binding of EGCG to αSN within the dead time is responsible for this signal disappearance, this suggests that αSN has a capacity to bind up to 54 EGCGs per αSN protomer in the formation of EGCG-induced oligomer. The appearance of EGCG-induced aggregates of αSN was monitored by transmission electron microscopy (TEM) (Fig. 2*D*), which revealed small round oligomers that resembled those observed in other studies (25, 39), either isolated or connected into chains. We have previously observed small amounts of oligomeric concatamers in the absence of EGCG, but not to the same extent as with EGCG, indicating that EGCG promotes formation of these high molecular weight assemblies.

**Figure 3:**
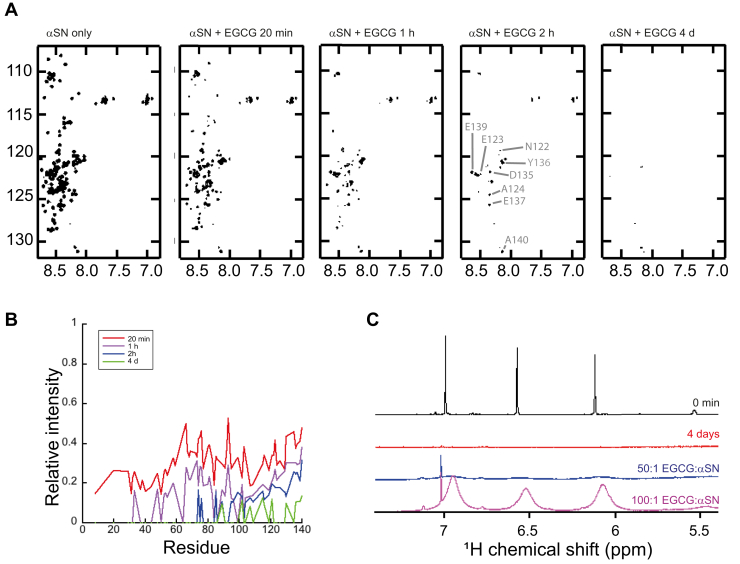
**Oligomerization of αSN in the presence of EGCG involves mainly the first 80 residues.***A*, ^15^N–^1^H HSQC of αSN upon incubation with EGCG ([αSN]:[EGCG] = 1:20) over time. *B*, relative intensity of peaks in ^1^H–^15^N HSQC spectra (*A*) of 0 min, 1 h, 2 h, and 4 days relative to αSN alone. *C*, 1D spectrum of 3 mM EGCG mixed with 150 μM αSN ([EGCG]:[αSN] = 20:1) immediately after mixing (*black*) and after 4 days of incubation (*red*). 28 mM EGCG was stepwise added to give first an [EGCG]:[αSN] of 50:1 (*blue*) and then 100:1 (*magenta*). The sharp peak at 7.1 ppm stems from an impurity in the EGCG sample. The assigned ^15^N–^1^H HSQC spectrum of αSN with high resolution is provided as Figure S4. αSN, α-synuclein; EGCG, (−)-epigallocatechin gallate; HSQC, heteronuclear single quantum coherence.

To investigate the oligomerization process with higher structural resolution, time-dependent signal loss was monitored by 2D ^15^N–^1^H heteronuclear single quantum coherence (HSQC) NMR spectroscopy for a 20:1 ligand-to-protein ratio. We observe severe signal loss for all protein residues already after 20 min (Fig. 3*A*). Based on the observations made at higher EGCG ratio, this observation is consistent with a scenario in which most αSN is sequestered into NMR-invisible oligomeric species, removing all free EGCGs from solution in a cooperative process. As there is insufficient EGCG to aggregate all protein within the first rapid phase (<10% of the signal disappears in the burst phase at 20:1 EGCG:αSN, *cf.* Fig. 2*C*; this fraction is likely higher in the HSQC experiment where we have a fivefold increase in αSN and EGCG concentration), the remaining αSN will be subject to slower self-association processes. Plotting signal intensity *versus* residue number reveals a second and slower loss of the NMR-visible pool that is not evenly distributed over the sequence (Fig. 3*B*). Resonances belonging to the N-terminal portion of the protein (residues 1–70) are completely lost after 2 h, whereas signals from the C-terminal domain (residues 95–140) still remain detectable after multiple days. Previous 2D ^15^N–^1^H HSQC NMR studies have shown that the C-terminal ∼40 residues remain highly mobile (and thus visible) in αSN oligomers that form in the absence of EGCG (25). In combination, these studies suggest the rapid (minutes) formation of EGCG-aggregated oligomers, followed by a slower (hours) phase producing oligomers in which only the C terminus remains flexible, and finally very slowly (days) forming oligomers with rigidified C termini. Although the kinetics differ from those seen in Figure 2 as the HSQCs were recorded at higher concentration, structural insight into oligomer formation was obtained in these experiments.

To analyze whether the αSN assemblies that formed during 4 days of incubation with EGCG at 20:1 were able to bind additional EGCG, we added EGCG to 50-fold and then to 100-fold excess in two steps and monitored the signal in the aromatic region, characteristic for EGCG (Fig. 3*C*). Only an extremely weak signal was obtained at 50-fold excess EGCG, consistent with the estimated ability of αSNs to bind around 54 EGCGs per protomer (Fig. 2*C*). At 100-fold EGCG, intense but broad peaks were observed, similar to the signals seen at 60-fold excess where EGCG was added to the αSN monomer in a single step (Fig. 1*B*). Thus, a fixed amount of EGCG is sequestered during oligomer formation, and excess-free EGCG is present in a dynamic binding equilibrium with the oligomeric assemblies. It is important to bear in mind that despite their broad appearance, the EGCG peaks belong to free ligand. The line broadening here is not because of slow tumbling but arises from kinetic exchange with large molecular weight species (33).

### EGCG binding to preformed oligomer

**Figure 4:**
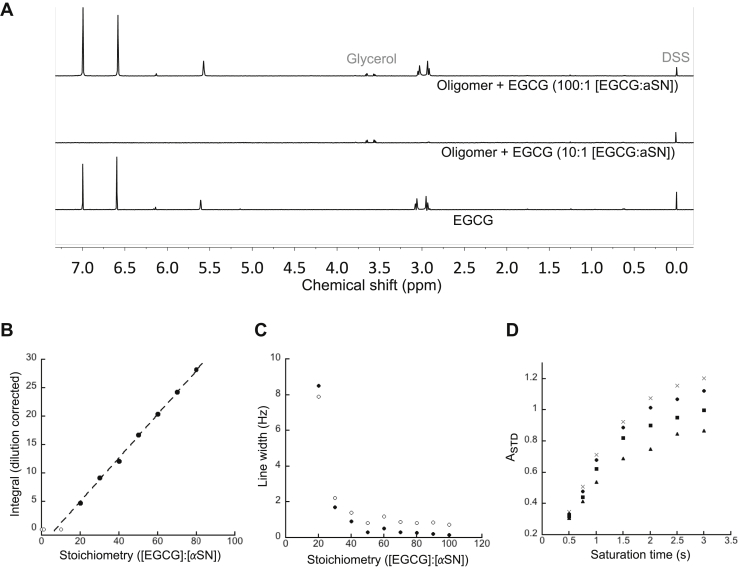
**Binding of EGCG to preformed oligomer.***A*, titration of EGCG to αSN oligomer. At 1:10 = [αSN]:[EGCG], all EGCG peaks are broadened beyond observation. The peaks around 3.6 ppm are due to a small glycerol contamination in the oligomer sample. At excess EGCG (1:100 [αSN]:[EGCG]), peaks from EGCG reappear. *B*, integration of H_2”,6”_ peak at different stoichiometries gives a binding stoichiometry of 7 ± 1 ([EGCG]:[αSN]) from the intercept at the *x*-axis. *C*, line width of 2”,6” (*filled circles*) and 2’,6’ peaks (*open circles*) of EGCG at the different stoichiometries. *D*, STD amplification factor. STD intensity as a function of saturation time at 1:100 ([αSN]:[EGCG]) (saturation field applied at −1 ppm) for 2”,6” (*crosses*), 2’,6’ (*circles*), 3 (*squares*), and 4_Eq_,4_Ax_ (*triangles*). αSN, α-synuclein; EGCG, (−)-epigallocatechin gallate; STD, saturation transfer difference.

Finally, we investigated the binding of EGCG to preformed αSN assemblies. To this end, αSN oligomers were preformed by incubating monomeric αSN at high concentrations (8–10 mg/ml) under orbital shaking at 37 °C for 3 h (*i.e.*, in the absence of EGCG) and separated by size-exclusion chromatography (25). This type of oligomer consists of ∼30 protomer units (11, 14). Subsequently, EGCG was titrated into NMR vials containing 50 μM (protomer concentration) oligomers, and 1D ^1^H-NMR spectra were recorded at each titration step. EGCG signals (here the integrated intensity of the EGCG H_2”,6”_ peak) only appeared at higher EGCG:αSN ratios (Fig. 4*A*), and their integral increased linearly (Fig. 4*B*). By extrapolating the EGCG signal back to zero integral (free ligand concentration), we determine a high-affinity binding interaction where 7 ± 1 EGCG molecules are immobilized per αSN protomer in the oligomeric assembly. EGCG signals at molar ratios below 20 were very broad and could not be accurately integrated. These are therefore not included in the extrapolation. A plot of line width *versus* stoichiometry (Fig. 4*B*) shows that high-affinity binding is followed by dynamic ligand exchange as described earlier for the monomeric form, that is, broad peaks at low ligand ratios and a sharpening at higher ratios (*cf.*, Figs. 1*B*, 3*C*, and 4*C*). This reflects that addition of more ligand increases the fraction of free ligand. In addition, higher EGCG concentrations lead to an increased on-rate, which is reflected in faster kinetics. As a result, peaks sharpen when EGCG concentration is increased (Fig. 4*C*). A small gradual downfield change in chemical shift is seen for the EGCG peaks at higher concentrations, which we attribute to weak self-association, possibly leading to covalent dimerization as mentioned previously. This shift is also observed in binding experiments with αSN monomers (Fig. 1*B*). Consistent with this, EGCG shows the same behavior on its own at higher concentrations (Fig. S3).

To confirm the dynamic interaction between free EGCG in solution with the oligomer directly, we turned to saturation transfer difference (STD) experiments. In these experiments, the oligomer magnetization is progressively saturated by a weak RF field applied to the protein methyl transitions. The slow tumbling of the oligomeric assembly makes the transfer of saturation through ^1^H–^1^H dipolar couplings (known as cross-relaxation) very effective, such that all protein signals become quenched. When a small molecule transiently binds during the saturation time, cross-relaxation will lead to partial attenuation of the ligand signal, depending on the lifetime of complex formation and the distance between ligand and protein hydrogens. A difference spectrum directly displays all ligand signals that experience saturation transfer from binding. As the amount of transfer depends on the “contact time” of ligand to protein, a series of increasing saturation times shows progressive transfer of magnetization from protein to ligand. We applied the STD experiment to the sample containing the largest excess of EGCG to oligomer ratio (100:1), where rapid binding kinetics and low-affinity binding prevail (*i.e.*, bound state lifetime <1 s; *K*_*d*_ ∼ μM–mM (40, 41)). Saturation curves for several hydrogen atoms of EGCG (Fig. 4*D*) directly demonstrate weak oligomer binding.

## Discussion

### EGCG drives αSN into oligomers of defined stoichiometry in a concerted manner

The small flavonoid polyphenol EGCG is able to promote the formation of nontoxic and unstructured αSN oligomers from both monomer αSN (16) and amyloid fibrils (22). Here, we have monitored EGCG-induced αSN oligomer formation by means of NMR signal loss of monomer over a fixed period. When monomeric αSN is incubated with EGCG at low stoichiometry ([EGCG]:[αSN] = 8.7), no change in the relative NMR signal intensities of αSN is observed over 10 h, indicating that all added αSN is present in solution. However, increasing the stoichiometry to 51.9:1 leads to the disappearance of all protein NMR signals together with those for EGCG. This suggests that, at short times and at a critical concentration, EGCG induces the rapid and comprehensive sequestration of αSN in a concerted manner. Incubation with more than 50-fold excess leads to the renewed observation of ligand signals, demonstrating that the oligomers comprise a well-defined amount of EGCG. Extrapolation leads to an [EGCG]:[αSN] stoichiometry of 54:1 for the saturated complex. Assuming that EGCG binds uniformly along the entire 140-residue polypeptide chain (our previous studies on the effect of EGCG on αSN peak intensities (18) indicated that all residues were affected, although two broad peak areas were identified in the N- and C-terminal regions, respectively), αSN sequesters one molecule of EGCG per 2.7 amino acids of disordered protein. Although the oligomeric state of αSN induced by EGCG is inaccessible to solution-state NMR spectroscopy, solid-state NMR and complimentary biophysical techniques indicate a disordered structure (8). We also show that, over time, EGCG-induced oligomers lose their flexible C terminus when incubated with sufficient (20:1) EGCG. Previous studies have shown EGCG-induced dose-dependent NMR line broadening for αSN at 5:1 and 10:1 [EGCG]:[αSN], *that is*, at slightly lower stoichiometries than we explore here (8). Although the kinetics cannot be directly compared because of differences in concentration and temperature to the present study, the observation that oligomers formed at 10:1 [EGCG]:[αSN] are heterogeneous in size and can still bind antibodies that recognize the flexible C domain, most likely result from the coexistence of a mixture of EGCG-saturated and immobilized oligomers and monomeric species. The data in Figures 2 and 3 are also in line with the solid-state NMR data of Fusco *et al.* (8), where the immobilized regions of protomers contribute to dipolar-assisted rotational resonance spectra, whereas those that present a flexible C terminus contribute to both dipolar-assisted rotational resonance and insensitive nuclei enhancement by polarization transfer spectra for the rigid and flexible parts of the sequence, respectively. Unfortunately, these spectra are insufficiently quantitative to extract relative populations. Furthermore, solid-state NMR studies of oligomers formed in the absence and presence of EGCG reveal structural differences in the core of the oligomer, but both types of oligomers contain a flexible C terminus (8). The fact that the N-terminal (residues 1-60) and non-amyloid β (NAC; residues 61-95) regions remain immobilized in the EGCG-induced oligomer along with the fact that oligomers formed in the presence of EGCG are nontoxic suggest that the NAC region and N terminus are important determinants for the toxicity of αSN oligomers (5) and that the binding of EGCG to the N terminus is able to abrogate this toxicity.

### EGCG-dependent oligomerization of αSN is graded and proceeds from N terminus to C terminus

In the presence of 20 equivalents of EGCG, αSN first undergoes a phase of oligomerization within few hours that is accompanied by a loss of intensity for all residues. This is followed by a second slower association phase that takes place over several days. We interpret the first phase to result from a cooperative EGCG-dependent formation of oligomers. At the same time, loss of signal at the N terminus is stronger, suggesting that parallel oligomer formation pathway exists. The species that are tethered at the N-terminal region approximately 50 residues slowly become NMR invisible over the following days. NMR signals of the C terminus eventually disappear over the course of several days, and this process is distinct from that observed for oligomers formed in the absence of EGCG, which are characterized by the persistence of disorder in the C-terminal region ∼40 amino acids over days to weeks (12). A possible explanation for these observations is that EGCG-induced oligomers can self-associate to longer chains, as demonstrated by TEM.

The graded response, with signal loss in first N-terminal then NAC region and at last C-terminal region, is highly reminiscent of that seen for αSN interaction with 1-palmitoyl-2-oleoyl-sn-glycero-3-phospho-(1′-rac-glycerol) in small unilamellar vesicles and nanodiscs (42, 43). However, αSN binding to negatively charged membranes forms alpha-helix structure, whereas oligomer formation proceeds without secondary structure formation.

The slope of two obtained from the power law relationship between the rate (and rate constant) of signal loss and EGCG stoichiometry (Fig. 2*B*) indicates that two EGCG molecules come together to react with αSN per encounter. Clearly, this does not limit the final stoichiometry of binding, which is more than an order of magnitude higher (54 EGCG:αSN). Rather, it implies that several EGCG molecules interact cooperatively when binding to αSN, perhaps by forming protein-stabilized dimers that increase affinity and thus the stability of the bound EGCG molecules. Other dimeric forms of EGCG bind to Aβ40 and bovine insulin and inhibit fibrillation more effectively than EGCG, most likely because of their ability to make a larger number of hydrophobic interaction and hydrogen bonds with the polypeptide chain (44, 45).

### EGCG binds to oligomers that are formed in the absence of EGCG

We obtained a binding stoichiometry of seven EGCGs per protomer of preformed oligomers based on a linear extrapolation of the free ligand NMR signal integral. This number is much smaller than the ∼54 EGCG molecules that associate with monomeric αSN during oligomer formation. The disparity indicates that multiple EGCG molecules are sequestered within the oligomer during its formation but cannot access this region once the oligomer is formed. Although the exact nature and sequence specificity of binding remain unclear (8), studies with other amyloidogenic proteins implicate aromatic sidechains (46, 47), binding of EGCG to αSN oligomers reduces the flexibility of the C terminus (which contains three Tyr and one Phe) (25), and EGCG has different binding modes dependent on the conformational state of the protein (17). As STD–NMR signals are typically obtained with macromolecule–ligand interactions with millimolar to micromolar binding constants (41), EGCG is expected to bind with weak affinity, in line with the fast-exchange behavior that is observed in titration experiments with monomeric αSN. Line broadening observed for excess EGCG in dynamic binding equilibrium is the hallmark of a ligand binding to large aggregates and also shows the expected narrowing when the excess ligand pool is increased, further corroborating the fluxional character of the interaction.

### EGCG reduces cellular toxicity well below oligomer conversion stoichiometry

Since αSN toxicity may be mitigated by the formation of nontoxic oligomers, or by inhibition of membrane binding, EGCG may be an interesting lead for therapeutics aimed against Parkinson's disease. For example, EGCG has been shown to inhibit extracellular toxicity toward OLN93 cells by 50% at [EGCG]:[αSN] = 0.36:1, and *in vitro* calcein release was likewise inhibited at [EGCG]:[αSN] = 0.23:1 (25). The present study suggests that, under these conditions, EGCG acts at levels several orders of magnitude below those required for oligomer conversion. Possibly, the cooperative nature of EGCG-induced conversion, along with the cooperation between EGCG molecules in the binding reaction demonstrated here, may effect the accumulation of populations of oligomers sufficiently to generate the required response.

## Experimental procedures

### Preparation of recombinant αSN

Recombinant human αSN was produced in *Escherichia coli* using a pET11-D αSN construct induced *via* autoinduction as described (11, 48). The same procedure was followed to produce uniformly ^15^N-labeled αSN, except that *E. coli* was grown in minimal medium using ^15^NH_4_Cl as the only nitrogen source.

### EGCG solution

EGCG (Sigma; 458.4 Da) was weighed out and dissolved in PBS (heavy water) in an Eppendorf tube to a final concentration of 10 mM. The tube was wrapped in an aluminum foil to protect the sample from light and stored at −20 °C between experiments. The solution was used within 1 week and was always transparent, showing no sign of oxidation.

### Oligomer preparation and purification

αSN oligomers were purified as described (9, 15) with the modification that the sample was incubated for 3 h under fibrillation conditions prior to purification. αSN was dissolved to 8 to 10 mg/ml and incubated at 37 °C, 900 rpm for 3 h. Insoluble material was removed by centrifugation at 4 °C at 13,000 rpm for 10 min, and the supernatant was loaded on a Superose 6 prep grade XK 26/100 column (GE Healthcare) equilibrated in PBS buffer. Oligomer fractions were collected and stored at 4 °C and concentrated using a 100 kDa spin filter that was washed with PBS to remove glycerol.

### NMR spectroscopy

NMR experiments were performed on deuterated samples at 278 K (αSN incubation with EGCG followed over time with HSQC; Fig. 3) or 283 K on Bruker Avance NMR spectrometers at ^1^H frequency of 950 MHz or 500 MHz. The spectra were processed using NMRPipe (49) and analyzed with Sparky (T. D. Goddard and D. G. Kneller; SPARKY 3, University of California, San Francisco), unless otherwise stated. Sodium salt of 2,2-dimethyl-2-silapentane-5-sulphonic acid (DSS) was included in all samples for chemical shift referencing (50).

#### Titration experiments

For titration of EGCG to monomer, separate samples were prepared. αSN was added to each sample right before the NMR measurement. The dead time from sample preparation to NMR experiment was 10 min. The samples contained 30 μM αSN monomer, 50 μM DSS, and 0, 0.15, 0.3, 0.6, 1.8, or 3 mM EGCG, in deuterated PBS. Reference spectra of EGCG were recorded for 0, 0.15, 0.3, 0.6, 1.0, 1.2, 2.5, 1.8, and 3 mM EGCG. For titration of EGCG to oligomer, an oligomer sample of 30 μM αSN oligomer 50 μM DSS in deuterated PBS is used. Freshly prepared 10 mM EGCG stock solution was added to the samples and mixed giving 1, 10, 20, 30, 40, 50, 60, 70, 80, 90, and 100 [EGCG]:[αSN]. 1D ^1^H-NMR employed water suppression using excitation sculpting with gradients and 32 scans for each experiment.

#### Time-dependent EGCG-induced αSN oligomerization followed by 1D ^1^H-NMR

Samples contained 34.7 μM αSN, 50 μM DSS, and 300, 600, 900, 1200, and 1800 μM EGCG (*i.e.*, EGCG:αSN molar ratios of 8.7, 17.3, 25.9, 34.6, and 51.9) in PBS. ^1^H-NMR employed water suppression using excitation sculpting with gradients with 128 scans for each experiment and variable number of dummy scans to change time between NMR experiments. The integrated methyl signal from αSN is normalized to the glycerol signal (contaminant in αSN sample) as an internal control. Exponential fit was prepared with MATLAB and varying only amplitude and velocity constant by setting the baseline to 0.05.

#### Time-dependent EGCG-induced αSN oligomerization followed by 2D ^15^N–^1^H HSQC

Samples contained 150 μM ^15^N-αSN, 80 μM DSS, and 3 mM EGCG in PBS. The matrix size of the HSQC was 128 (t_1_) and 1024 (t_2_) complex points, with a spectral width of 32 and 16 ppm for the ^15^N and ^1^H dimensions, respectively. A gradient and sensitivity-enhanced version of the HSQC experiment was used that is optimized to avoid mixed-phase artifacts (51).

#### 1D STD NMR

About 35.7 μM of unlabeled oligomer incubated with 3.57 mM EGCG (1:100) was monitored by acquiring 1D STD NMR spectra (52). STD spectra were recorded with 64 scans, 32 K complex points, and a spectral width of 16.04 ppm. Selective saturation of αSN oligomers was achieved through methyl proton irradiation at −1 ppm using a Gaussian-shaped pulse train of 50 ms. A 30 ms spin lock (T_2_ filter) was employed to suppress protein signals. Off-resonance (reference) experiment was recorded with saturation at 42 ppm. Spectra with 0.5, 0.75, 1, 1.5, 2, 2.5, and 3 s saturation times were recorded. For each saturation time, four spectra were recorded, alternating on and off resonance. The STD amplification factor *A*_STD_ was calculated for each saturation time. *A*_STD_ was calculated as $A_{\text{STD}}=\frac{I_{0}-I_{\text{sat}}}{I_{0}}*\frac{\left[L\right]}{\left[P\right]}$, where *I*_0_ is the intensity in the reference spectrum and *I*_sat_ is the intensity in the spectrum with irradiation at −1 ppm, [L] is the total EGCG concentration, and [P] is the αSN concentration. Signals of protons (2’,6’), (2”,6”), 3, and 4 (axial and equatorial) were followed. See Fig. S1 for assignments.

### TEM

Samples were taken from NMR samples after time-dependent experiment at the indicated stoichiometries and transferred to a 400-mesh carbon-coated copper grid (EM resolution) that was glow discharged for 30 s. The oligomer sample was transferred to the grids and washed on two drops of doubly distilled water, stained with 1% uranyl formate, and blotted dry on filter paper. The samples were viewed at 120 kV. EM was done on a Tecnai G2 Spirit (FEI Company), and images were taken using a TemCam F416 camera (TVIPS).

## Data availability

Data can be shared upon request to the corresponding author.

## Supporting information

This article contains supporting information.

## Conflict of interest

The authors declare that they have no conflicts of interest with the contents of this article.
