# Supplementary material for: How epigallocatechin gallate binds and assembles oligomeric forms of human alpha-synuclein
Source: J Biol Chem. 2021 May 18;296:100788. doi: 10.1016/j.jbc.2021.100788 (PMC8191297; doi:10.1016/j.jbc.2021.100788)
Supplement: Supplemental Figures S1–S4 [file mmc1.docx]

**Supporting information**

**How epigallocatechin gallate binds and assembles oligomeric forms of human alpha-synuclein**

Camilla B. Andersen^1,2^, Yuichi Yoshimura^1,3,5^, Janni Nielsen^1^, Daniel E. Otzen^1,4*^, Frans A. A. Mulder^1,5*^

^1^ Interdisciplinary nanoscience center (iNANO), Gustav Wieds Vej 14, Aarhus University, 8000 Aarhus C, Denmark

^2^ Present address: Department of Chemical Engineering, Biotechnology and Environmental Technology, University of Southern Denmark, Campusvej 55, 5230 Odense, Denmark

^3^ Present address: Institute for Protein Research, Osaka University, Yamada-oka 3-2, Suita 565-0871, Japan

^4^ Department of Molecular Biology and Genetics, Gustav Wieds Vej 10C, Aarhus University, 8000 Aarhus C, Denmark

^5^ Department of Chemistry, Langelandsgade 140, Aarhus University, 8000 Aarhus C, Denmark

* To whom correspondence should be addressed: [dao@inano.au.dk](mailto:dao@inano.au.dk); fmulder@chem.au.dk

**
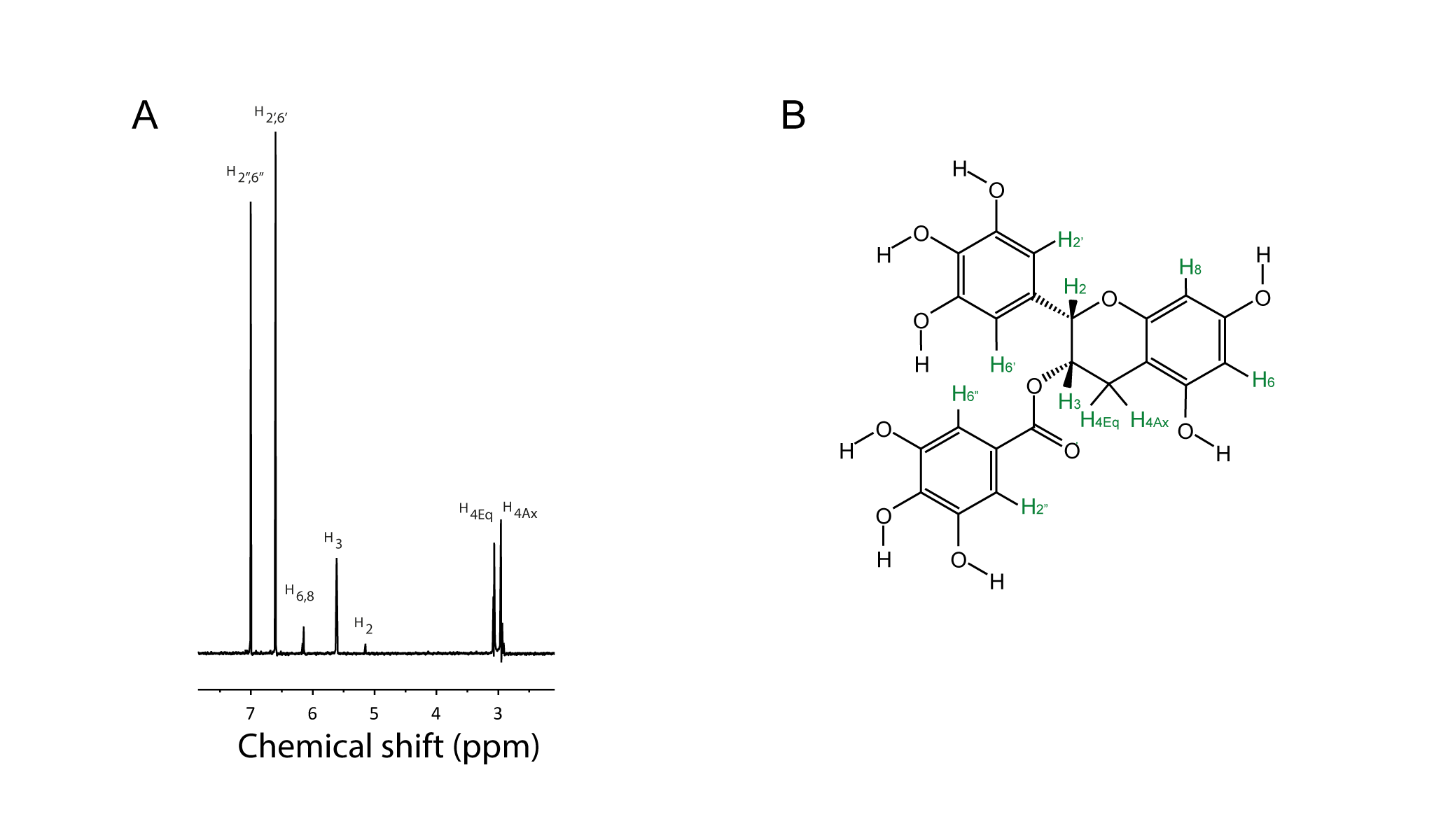
**

***Figure S1:*** Spectral and structural properties of EGCG. A: 1D ^1^H-NMR spectrum of EGCG in PBS, 100% D_2_O. B: Structure of EGCG. Protons observed in the NMR spectrum are shown in green.

**
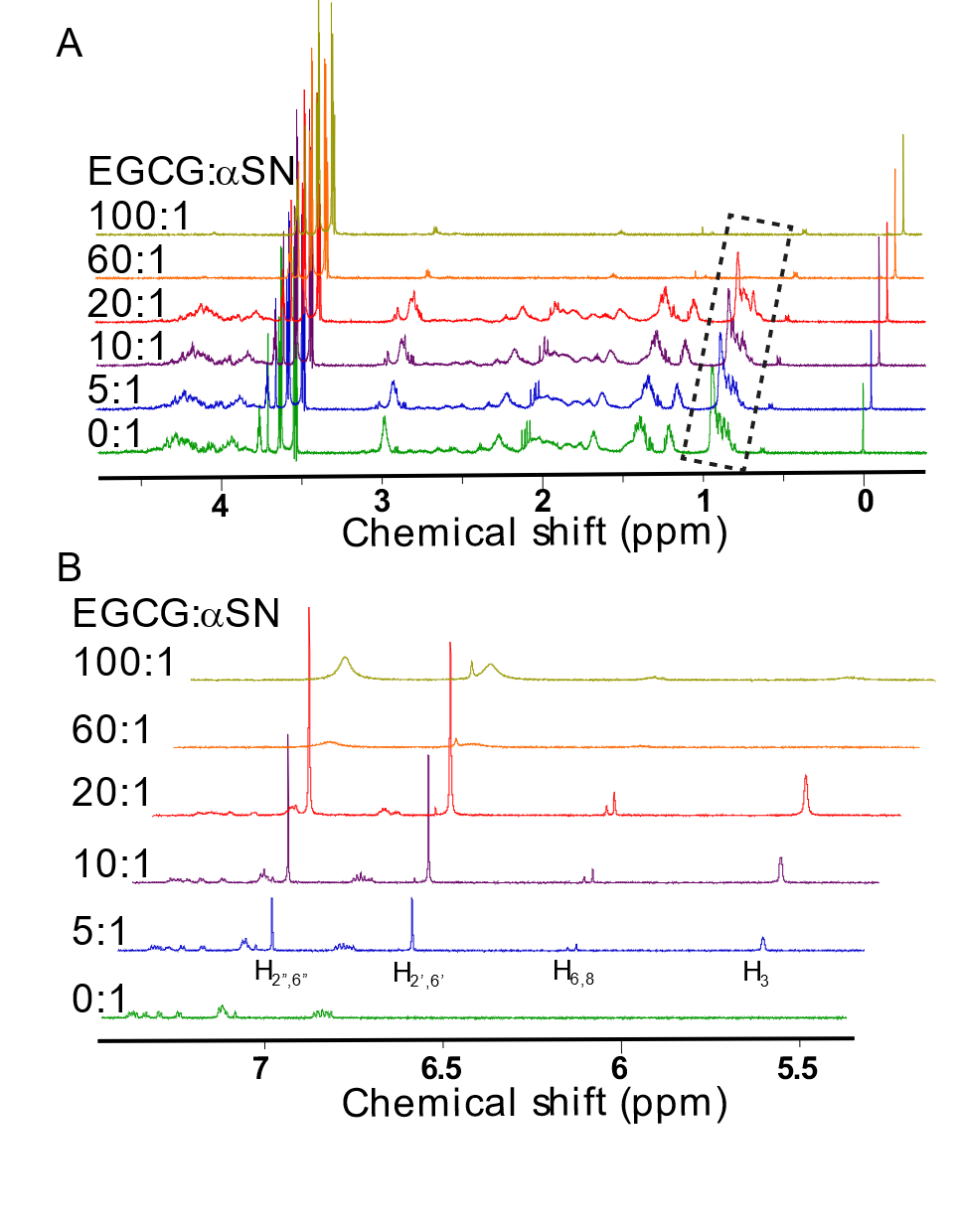
**

***Figure S2:*** 1D ^1^H-NMR of αSN and EGCG at different stoichiometries (unedited version of figure 1A and 1B). (A) The methyl region from αSN (dotted box). (B) The aromatic region (dominated by EGCG - H_2”,6”_, H_2’,6’_, H_6,8_ and H_3_ are assigned). For visibility, the spectra in A and B are offset by 0.05 ppm for each trace.

**
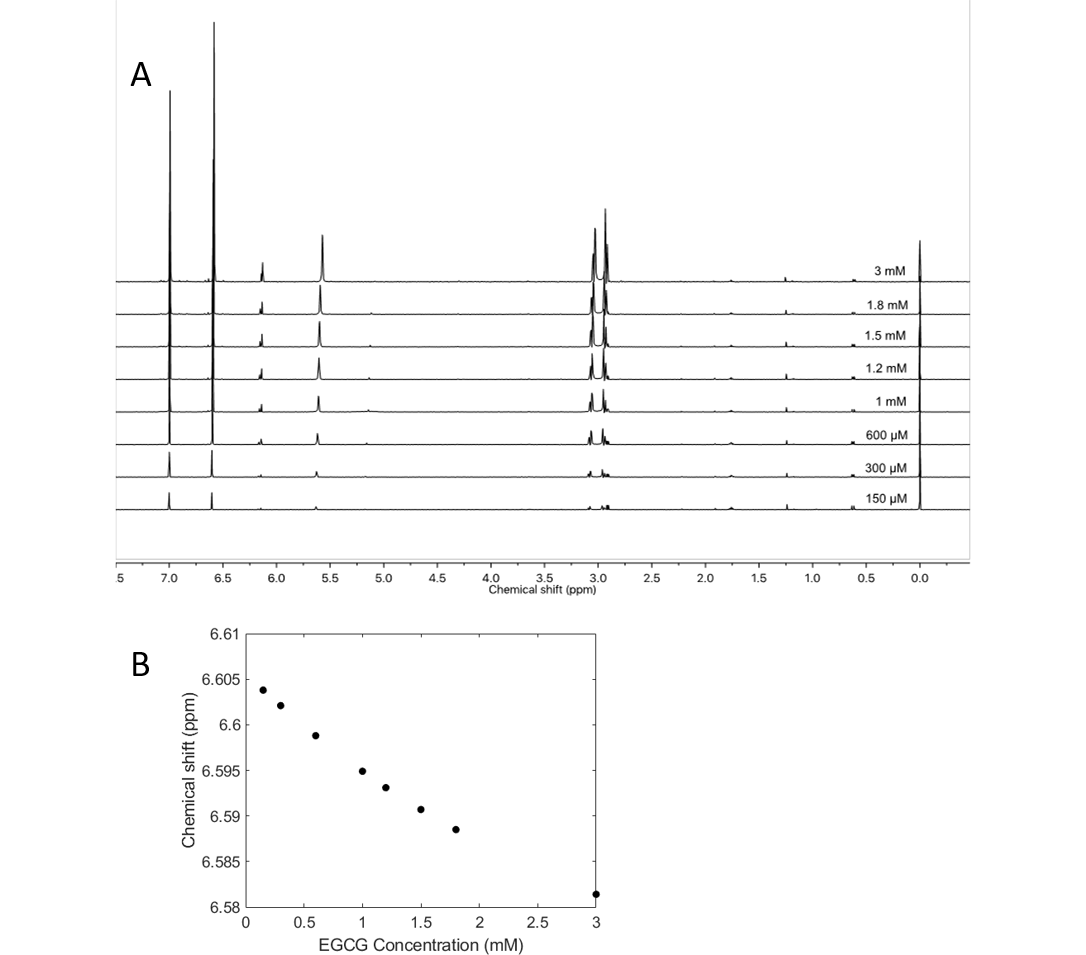
**

***Figure S3***: (A) 1D ^1^H-NMR spectra of 0.15-3 mM EGCG. (B) Chemical shift of H_2’,6’_ (at 6.6 ppm) as a function of concentration illustrates the chemical shift dependent on EGCG concentration.

**
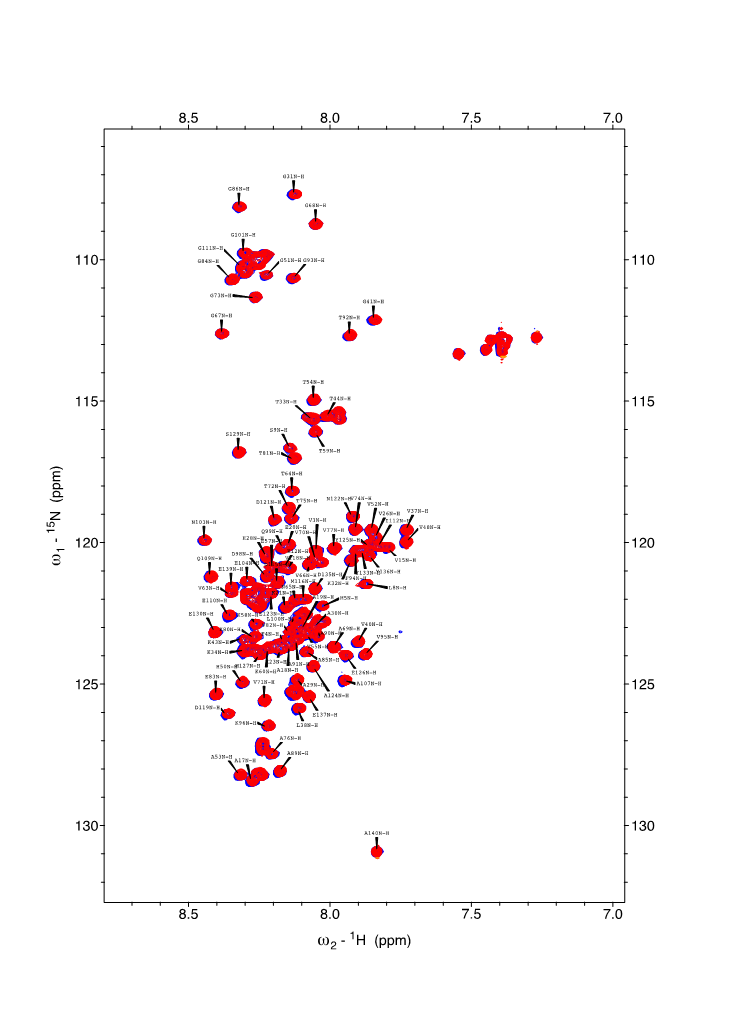
**

***Figure S4:*** HSQC spectrum of ^15^N-αSN recorded in the absence (blue) and in the presence of EGCG ([EGCG]:[αSN] = 2:1) (red) after 10 hr. incubation at 283K.
